# *Clostridioides difficile* Toxin CDT Induces Cytotoxic Responses in Human Mucosal-Associated Invariant T (MAIT) Cells

**DOI:** 10.3389/fmicb.2021.752549

**Published:** 2021-12-21

**Authors:** Isabel Marquardt, Josefine Jakob, Jessica Scheibel, Julia Danielle Hofmann, Frank Klawonn, Meina Neumann-Schaal, Ralf Gerhard, Dunja Bruder, Lothar Jänsch

**Affiliations:** ^1^Cellular Proteomics, Helmholtz Centre for Infection Research, Braunschweig, Germany; ^2^Institute of Medical Microbiology and Hospital Hygiene, Infection Immunology, Health Campus Immunology, Infectiology and Inflammation, Otto-von-Guericke University Magdeburg, Magdeburg, Germany; ^3^Immune Regulation, Helmholtz Centre for Infection Research, Braunschweig, Germany; ^4^Braunschweig Integrated Centre of Systems Biology (BRICS), Department of Bioinformatics and Biochemistry, Technical University Braunschweig, Braunschweig, Germany; ^5^Metabolomics, Leibniz Institute DSMZ-German Collection of Microorganisms and Cell Cultures, Braunschweig, Germany; ^6^Institute of Toxicology, Hannover Medical School, Hannover, Germany

**Keywords:** MAIT cells, *C. difficile*, TcdA, TcdB, CDT, CDAC, IL-18, MR1-mediated MAIT cell activation

## Abstract

*Clostridioides difficile* is the major cause of antibiotic-associated colitis (CDAC) with increasing prevalence in morbidity and mortality. Severity of CDAC has been attributed to hypervirulent *C. difficile* strains, which in addition to toxin A and B (TcdA, TcdB) produce the binary toxin *C. difficile* transferase (CDT). However, the link between these toxins and host immune responses as potential drivers of immunopathology are still incompletely understood. Here, we provide first experimental evidence that *C. difficile* toxins efficiently activate human mucosal-associated invariant T (MAIT) cells. Among the tested toxins, CDT and more specifically, the substrate binding and pore-forming subunit CDTb provoked significant MAIT cell activation resulting in selective MAIT cell degranulation of the lytic granule components perforin and granzyme B. CDT-induced MAIT cell responses required accessory immune cells, and we suggest monocytes as a potential CDT target cell population. Within the peripheral blood mononuclear cell fraction, we found increased IL-18 levels following CDT stimulation and MAIT cell response was indeed partly dependent on this cytokine. Surprisingly, CDT-induced MAIT cell activation was found to be partially MR1-dependent, although bacterial-derived metabolite antigens were absent. However, the role of antigen presentation in this process was not analyzed here and needs to be validated in future studies. Thus, MR1-dependent induction of MAIT cell cytotoxicity might be instrumental for hypervirulent *C. difficile* to overcome cellular barriers and may contribute to pathophysiology of CDAC.

## Introduction

*Clostridioides difficile* infections (CDI) are the major cause of antibiotic-associated colitis and an emerging threat for hospitalized patients. In general, clinical symptoms including pseudomembranous colitis are associated with *C. difficile* toxin A (TcdA) and toxin B (TcdB; Pothoulakis, 1996; Chumbler et al., 2016). So-called hypervirulent *C. difficile* ribotypes that in addition to TcdA and TcdB express the binary toxin *C. difficile* transferase (CDT) are responsible for outbreaks and cause severe *C. difficile*-associated colitis (CDAC) with increased recurrence, morbidity, and mortality (Kuijper et al., 2008; Bacci et al., 2011; He et al., 2013). The presence of *C. difficile* toxins in the blood (toxemia) was linked to severe courses of CDI in animals and improved techniques for the detection of toxemia in CDI patients are being established (Steele et al., 2012; Yu et al., 2015).

*C. difficile* toxins introduce post-translational modifications to host signaling components following their endocytosis together with different surface receptors. TcdA and TcdB are glucosyltransferases, which post-translationally modify and inactivate GTPases in target cells resulting in breakdown of tight junctions and epithelial integrity (Just et al., 1995a,b; Feltis et al., 2000). In contrast to monomeric TcdA and TcdB, CDT consists of two subunits, the substrate binding component CDTb and the enzymatic component CDTa that ADP-ribosylates actin and thereby inactivates host cell actin polymerization, ultimately leading to increase of cell surface protrusion (Gülke et al., 2001). These toxin-induced modulations of host cell organization facilitate successful *C. difficile* colonization at the intestinal epithelium (Schwan et al., 2009, 2014; Kasendra et al., 2014; Cowardin et al., 2016a; Buccigrossi et al., 2019). Toxin-dependent effects on innate immune response in CDI include the induction of pro-inflammatory cytokine production, apoptosis of epithelial cells and macrophages and subsequent attraction of pro-inflammatory neutrophils that are major drivers of CDAC-specific immunopathology (Pothoulakis et al., 1988; Mahida et al., 1996; Rocha et al., 1997; Souza et al., 1997; Saavedra et al., 2018). While there is evidence that *C. difficile* toxins affect migration and chemotaxis of conventional T cells (Wu et al., 2013), it remains largely elusive to date whether and how these toxins interfere with functionally distinct T cell subsets. In this context, we demonstrated previously that compared to less toxigenic strains, hypervirulent *C. difficile* trigger superior responses in human mucosal-associated invariant T (MAIT) cells (Bernal et al., 2018).

MAIT cells constitute up to 10% of total T cells in the lamina propria (Treiner et al., 2003). They express high levels of the C-type lectin CD161 and the T cell receptor (TCR) α-chain Vα7.2 (Tilloy et al., 1999; Martin et al., 2009). This semi-invariant TCR, together with a limited TCRβ repertoire, restricts them to the major histocompatibility complex (MHC) class I-related protein MR1, which is expressed on antigen presenting cells and epithelial cells (Treiner et al., 2003; Le Bourhis et al., 2010; Dusseaux et al., 2011). MR1 presents riboflavin (vitamin B2)-derived metabolite variants that constitute a recently described class of antigens (Kjer-Nielsen et al., 2012; Corbett et al., 2014). Upon TCR stimulation, MAIT cells are able to immediately execute effector functions (Dusseaux et al., 2011). Beside the semi-invariant TCR, MAIT cells constitutively express IL-12 and IL-18 receptors enabling their cytokine-mediated activation (Ussher et al., 2014). Activated MAIT cells can mediate cytotoxicity by release of lytic granules containing effector molecules such as perforin and a set of granzymes, e.g., granzyme B (Kurioka et al., 2015). However, MAIT cells can as well execute inflammatory Th1 immunity for instance by interferon-γ (IFNγ) secretion. Previous reports have already demonstrated that MAIT cells can be activated by superantigenic toxins produced by *Staphylococcus aureus* and *Streptococcus pyogenes*, resulting in a substantial cytokine response (Shaler et al., 2017; Emgård et al., 2019).

Here, we have studied the responsiveness of peripheral human MAIT cells to *C. difficile* toxins and identified CDT to be most competent in inducing a cytotoxic effector phenotype in MAIT cells. Interestingly, CDT-induced MAIT cell responses depend on both the interleukin-18 (IL-18) and the MR1-dependent signaling pathway. We identified monocytes to be involved in MR1-dependent activation of MAIT cells following CDT stimulation. Data obtained in frame of this study suggest that MAIT cell cytotoxicity might contribute to clearance of toxemia or to pronounced immunopathology observed in CDAC that is caused by hypervirulent *C. difficile.*

## Materials and Methods

### *C. difficile* Cultures

*C. difficile* clinical isolates were provided by Leibniz Institute DSMZ – German Collection of Microorganisms and Cell Cultures (Braunschweig). DSM 102859 (RT023; depositor: Lutz von Müller) strain was cultured in riboflavin-free casamino acids containing medium (CDMM) under anaerobic conditions (Neumann-Schaal et al., 2015; Riedel et al., 2017). Cells were harvested at the mid exponential phase (OD_max_ 0.5). Bacterial numbers were determined using a Neubauer improved counting chamber (C-Chip, NanoEnTek). Bacterial cell pellets were harvested by centrifugation (13.000 *g*, 10 min, 4°C) and fixed with 2% paraformaldehyde (PFA) solution, were washed three times with PBS and stored at −80°C. Prior PBMC stimulation, the bacterial cells were resuspended in PBS to a final concentration of 3 × 10^8^ bacteria/ml.

### Expression and Purification of Recombinant *C. difficile* Toxins

Recombinant TcdA and TcdB as well as their glucosyltransferase deficient mutants were produced in the Gram positive and LPS-free *Bacillus megaterium* expression system (MoBiTec, Germany) as described before (Burger et al., 2003). Coding sequences for strain VPI10463 TcdA and TcdB were cloned into a modified pWH1520 vector (TcdA, TcdA D285/287 N) or into pHIS1522 vector (TcdB, TcdB D286/288 N) to obtain C-terminal His6-tagged proteins. The glucosyltransferase deficient mutants (TcdA D285/287 N, TcdB D286/288 N) were generated by site directed mutagenesis (QuikChange II Site-Directed Mutagenesis Kit, Stratagene) according to supplied protocol. For expression of proteins the *B. megaterium* strain WH320 was used. In contrast to the large clostridial glucosyltransferases both components of the binary toxin CDT (CDTa and CDTb from *C. difficile* strain R20291) were expressed in *E. coli* TG1. The coding sequence for mature CDTa, lacking the export sequence, was cloned into pQE30 vector for expression as N-terminally His6-tagged protein. Mature CDTb was expressed in *E. coli* as GST fusion protein using the pGEX2T vector (GE Healthcare) as described earlier (Beer et al., 2018). GST-CDTb was purified *via* glutathione (GSH)-sepharose columns according to standard protocol. After elution from GSH-sepharose beads the GST-CDTb fusion protein was activated by limited digestion with trypsin (0.2 μg trypsin per μg GST-CDTb) for 30 min at room temperature. Thereby, the GST-tag as well as the N-terminal inhibitory part of CDTb was removed, resulting in activated CDTb. Incubation with trypsin was terminated by addition of 2 mM 4-(2-Aminoethyl)-benzensulfonylfluorid (AEBSF). All His6-tagged proteins were purified *via* Ni2 + -NTA sepharose (Protino Ni-IDA columns, Macherey-Nagel, Germany). After buffer exchange *via* ZEBA desalting columns (Pierce/Thermofischer, Germany) all toxins were stored in 20 mM Tris–HCl, pH7.2, and 50 mM NaCl at −80°C. The specific concentration and purity of toxins was estimated by SDS gel electrophoresis. We have produced CDT as authors of this study have described before in Stieglitz et al. (2021). This report provides evidence by 1,000 CDT-regulated phosphorylation sites that CDT used in this study is LPS-free and did not induce any classical LPS-dependent signaling pathway such as the ERK pathway [Supplementary Tables 1 and 2 in Stieglitz et al. (2021)].

### Blood Donations

This study was conducted in accordance with the rules of the Regional Ethics Committee of Lower Saxony, Germany and the declaration of Helsinki. Buffy coats from blood donations of healthy human volunteers, who provided informed consent, were obtained from the Institute for Clinical Transfusion Medicine, Klinikum Braunschweig, Germany. Blood donors’ health was assessed prior blood donation. This procedure also includes standardized laboratory tests for infections with HIV1/2, HBV, HCV and *Treponema pallidum* (serology and/or nucleic acid testing) and hematological cell counts.

### PBMC Isolation and Stimulation

Buffy Coats were produced from whole blood donations by using the Top & Bottom Extraction Bag System (Polymed Medical Devices). Peripheral blood mononuclear cells (PBMCs) were isolated from buffy coats by Ficoll® Paque PLUS density gradient centrifugation (GE Healthcare GmbH). PBMCs were rested overnight in RPMI 1640 medium (Gibco/Life Technologies) supplemented with 10% fetal bovine serum gold (PAA Laboratories), 2 mM L-glutamine, 50 units/ml penicillin and 50 μg/ml streptomycin (all Gibco/Life Technologies) at 37°C in a humid 7.5% CO_2_ atmosphere. 0.5 × 10^6^ PBMCs were either left untreated or stimulated with PFA-fixed bacteria at different multiplicities of infection (MOI) and/or with 100 ng/ml *C. difficile* toxins for 20 h at 37°C. To validate the role of MR1 or IL-18, PBMCs were incubated with 5 μg/ml anti-IL18 (MBL International, clone 126-2H), 20 μg/ml anti-MR1 (Biolegend, clone 26.5), 20 μg/ml Isotype control (Biolegend, Mouse IgG1, κ isotype) or different concentrations of Ac-6-FP (Cayman Chemical) 1 h prior stimulation with CDT at 37°C. CDT was then added with 100 ng/ml and cells were incubated for 20 h at 37°C. For intracellular staining, Brefeldin A was added for the last 4 h of stimulation.

### Quantification of Cytokines

0.5 × 10^6^ PBMCs were left either untreated or stimulated in duplicates with fixed *C. difficile* bacteria at MOI 1 or with 100 ng/ml recombinant *C. difficile* toxins for 20 h at 37°C in duplicates. Supernatants of respective samples were pooled and enzyme-linked immunosorbent assays were performed to detect human IL-12 using Human IL-12 (p70) ELISA MAX™ kit (BioLegend), human IL-15 using Human IL-15 ELISA MAX™ kit (BioLegend), and human IL-18 using Human IL-18 ELISA kit (MBL).

### Fluorescence-Activated Cell Sorting (FACS) of MAIT Cells and Stimulation With *C. difficile* Toxins

PBMCs were isolated as described above. MAIT cells were sorted as CD3^+^ Vα7.2^+^ CD161^++^ lymphocytes using a FACSAria II flow cytometer (BD Biosciences; Bionozzle size: 70 μm; system pressure: 70 PSI; flow rate 30,000 events/s; laser: 488 nm with 100 mWatt for FITC, PE-Cy5, and PE-Cy7, 640 nm with 60 mWatt for APC; detection with bandpass filters for FITC 525/50, PE-Cy5 670/14, PE-Cy7 780/60, and APC 670/30). Sorted cells were washed with FACS buffer and rested overnight in RPMI 1640 medium (Gibco/Life Technologies) supplemented with 10% fetal bovine serum gold (PAA Laboratories), 2 mM L-glutamine, 50 units/ml penicillin and 50 μg/ml streptomycin (all Gibco/Life Technologies) at 37°C in a humid 7.5% CO_2_ atmosphere. 20,000 MAITs were either left untreated or stimulated with 100 ng/ml *C. difficile* toxins for 20 h at 37°C. For bi-cellular stimulation, monocytes were sorted as CD14^+^ cells (BV510, Biolegend, clone M5E2) and washed as described for MAIT cells. Directly after sorting, 20,000 monocytes were combined with 20,000 MAIT cells per well and treated with *C. difficile* CDT as described before. 5-OP-RU was used as a positive control and was kindly provided by Dr. Olivier Lantz (Institut Curie, Paris). 5-OP-RU was added for 20 h to the co-culture of MAIT cells and monocytes. As in other bicellular studies, MAIT cells were stimulated by using a concentration of 50 ng/ml 5-OP-RU, which induces efficient pro-inflammatory responses while maintaining viability of MAIT cells (Hagel et al., 2020). For intracellular staining, Brefeldin A was added for the last 4 h of stimulation.

### Antibodies

For cytometric assessment of MAIT cell phenotype, bulk PBMCs were stained with LIVE/DEAD™ Fixable Blue Dead Cell Stain Kit (Invitrogen) or Annexin V Apoptosis Detection Kit with 7-AAD FITC (Biolegend), with Fc receptor blocking reagent (Miltenyi Biotec) and a combination of the following antibodies (from BioLegend except as noted): CD3 BV605 (clone OKT3), CD161 APC (clone DX12, BD Biosciences), Vα7.2 PE-Cy7 (clone 3C10), CD69 PE (clone FN50), CD107a PerCP-Cy5.5 (clone H4A3), GzmB Pacific Blue (clone GB11), perforin FITC (clone), and IFNγ APC-Cy7 (clone 4S.B3). For MR1 expression analysis on antigen presenting cells, bulk PBMCs were stained with Fc receptor blocking reagent (Miltenyi Biotec), LIVE/DEAD™ Fixable Near-IR Dead Cell Stain Kit (Invitrogen), and a combination of the following antibodies (from BioLegend): CD11c BV421 (clone 3.9), CD123 BV510 (clone 6H6), CD14 BV421 (clone 3D3), CD3 BV605 (clone OKT3), CD45 FITC (clone 2D1), CD56 BV711 (clone 5.1H11), CD16 APC (clone 3G8), HLA-DR PE-Cy7 (clone LN3), and MR1 PE (clone 26.5).

### Extracellular and Intracellular Cell Staining

Stimulated PBMCs were washed with PBS and stained with Fc receptor blocking reagent (Miltenyi Biotec) and LIVE/DEAD™ Fixable Blue/Near-IR Dead Cell Stain Kit (Invitrogen). Cells were washed with FACS buffer and stained for extracellular surface marker at 4°C for 30 min. MR1 surface staining was performed together with the staining for extracellular surface marker. Cell fixation and permeabilization were performed using BD Cytofix/Cytoperm™ (BD Biosciences) followed by intracellular staining for 30 min at 4°C. Cells were washed twice with permeabilization buffer and cell pellet was resuspended in FACS buffer and subsequently analyzed on BD LSR-II SORP and BD LSR-Fortessa flow cytometer. Data was analyzed by FlowJo (TreeStar, v10.4.2) and Prism (GraphPad Software, v7.0c). To determine significant differences, Wilcoxon matched-pairs signed rank test was used. All *p*-values were corrected by strict Bonferroni Holm correction.

## Results

### Toxins of Hypervirulent *C. difficile* Activate Human MAIT Cells

**Figure 1:**
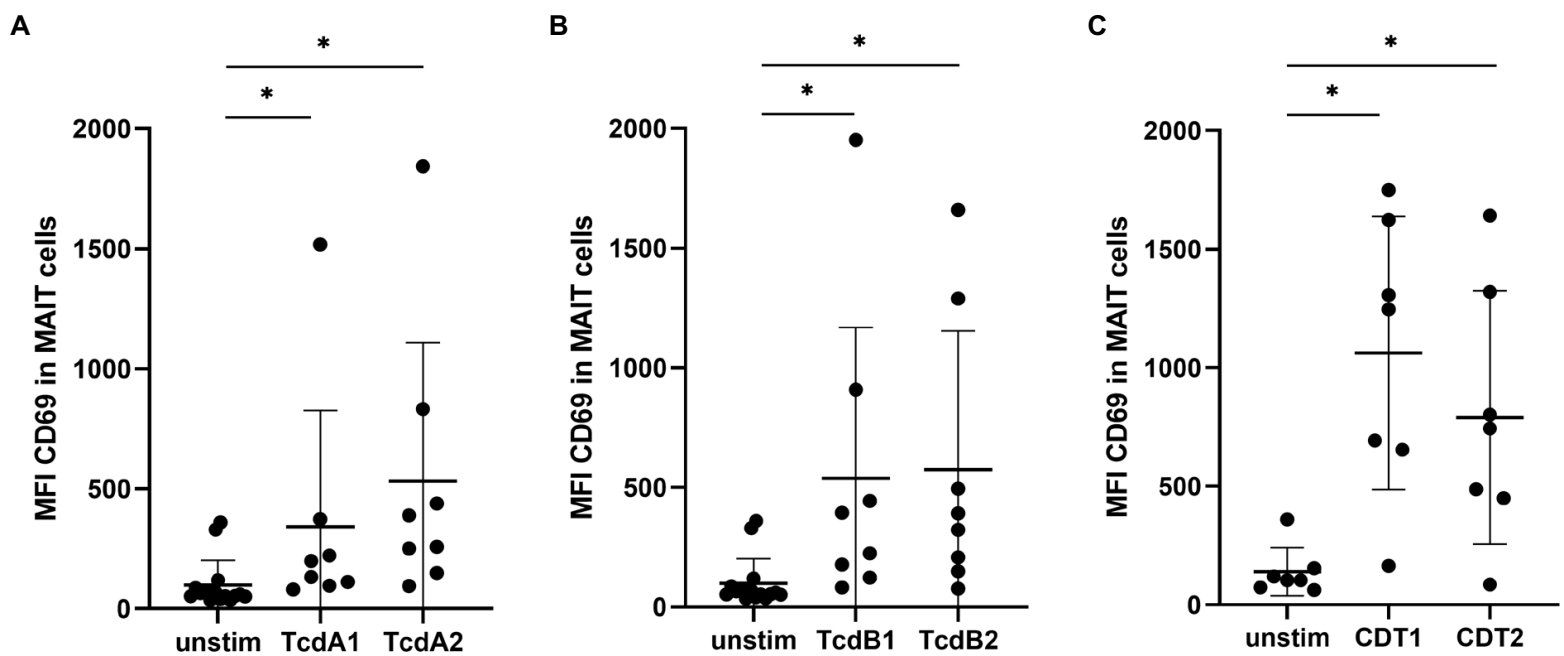
*C. difficile* toxin-induced MAIT cell activation. PBMCs were isolated from healthy donors and stimulated with 100 ng/ml *C. difficile* toxin from two different production batches. TcdA1 or TcdA2 **(A)**, TcdB1 or TcdB2 **(B)** and CDT1 or CDT2 **(C)** stimulation for 20 h followed by flow cytometric analysis of mean fluorescence intensity (MFI) of total CD69 of MAIT cells. MAIT cells were gated on CD161^++^ Vα7.2^+^ CD3^+^ T cells. Data represent two to four independent experiments from seven to eight donors. Horizontal lines indicate mean ± SD. Asterisks indicate significant differences as determined by three Wilcoxon matched-pairs signed rank tests between all given conditions with Bonferroni Holm correction: *p < 0.05.

We previously demonstrated that hypervirulent *C. difficile* bacteria, which express the toxins TcdA, TcdB and CDT, exhibit superior capacities than less toxigenic TcdA/B^+^ RT012 to activate human MAIT cells (Bernal et al., 2018). To further define the individual contribution of these major virulence factors to the observed MAIT cell activation, we generated recombinant TcdA, TcdB, their glucosyltransferase-deficient variants (GT-dTcdA and GT-TcdB), and binary *C. difficile* transferase (CDT) and studied their capacities to activate MAIT cells (purity of toxins shown in Supplementary Figure 1; gating strategy shown in Supplementary Figure 2). To this end, peripheral blood mononuclear cells (PBMCs) from healthy individuals were stimulated with increasing concentrations of CDT for 20 h from 1 to 100 ng/ml. Despite relatively pronounced donor variations, indeed all concentrations of CDT induced a significant increase in CD69 expression on MAIT cells, whereas the concentration of 100 ng/ml of CDT induced the highest MAIT cell activation (Supplementary Figure 3A). This concentration has also been shown (i) to induce effective innate immune response, (ii) to have no significant cytotoxic effect on the PBMC fraction, and (iii) to be within the range of concentrations present in fecal specimen of CDI patients (Lee et al., 2009; Ryder et al., 2010; Cowardin et al., 2016b; Yu et al., 2017). Therefore, we decided to investigate the role of the *C. difficile* virulence factors on MAIT cell activation using the concentration of 100 ng/ml. Interestingly, at 100 ng/ml not only CDT, but also TcdA and TcdB induced MAIT cell activation as indicated by a significantly increased CD69 expression (Figures 1A–C).

MAIT cell activation by TcdA and TcdB was further analyzed using glycosyltransferase-deficient TcdA (GT-dTcdA) or TcdB (GT-dTcdB). Both glycosyltransferase-deficient toxins induced a lower CD69 expression than their enzymatically active counterparts, indicating a glycosyltransferase-dependent mechanism (Supplementary Figure 4).

In contrast to TcdA and TcdB, CDT-mediated MAIT cell activation appeared to be less dependent on its enzymatic activity since the stimulation with the binding component CDTb was sufficient to induce full MAIT cell activation even in the absence of the enzymatic component CDTa. Adding increasing concentrations of CDTa to a fixed concentration of CDTb did not further increase MAIT cell activation (Supplementary Figures 3B,C). In contrast, adding increasing concentrations of CDTb to a defined concentration of CDTa indeed resulted in increased MAIT cell activation. This further emphasized the important role of the binding component CDTb in the mechanism of MAIT cell activation.

In conclusion, we found all three *C. difficile* toxins to activate human MAIT cells whereby CDT most effectively activated MAIT cells by its pore-forming unit CDTb. CDT is the hallmark of hypervirulent *C. difficile* strains and was therefore the focus in our following studies on endotoxin-mediated MAIT cell activation.

### CD161^++^ Vα7.2^+^ T Cells Are Efficiently Activated by CDT

**Figure 2:**
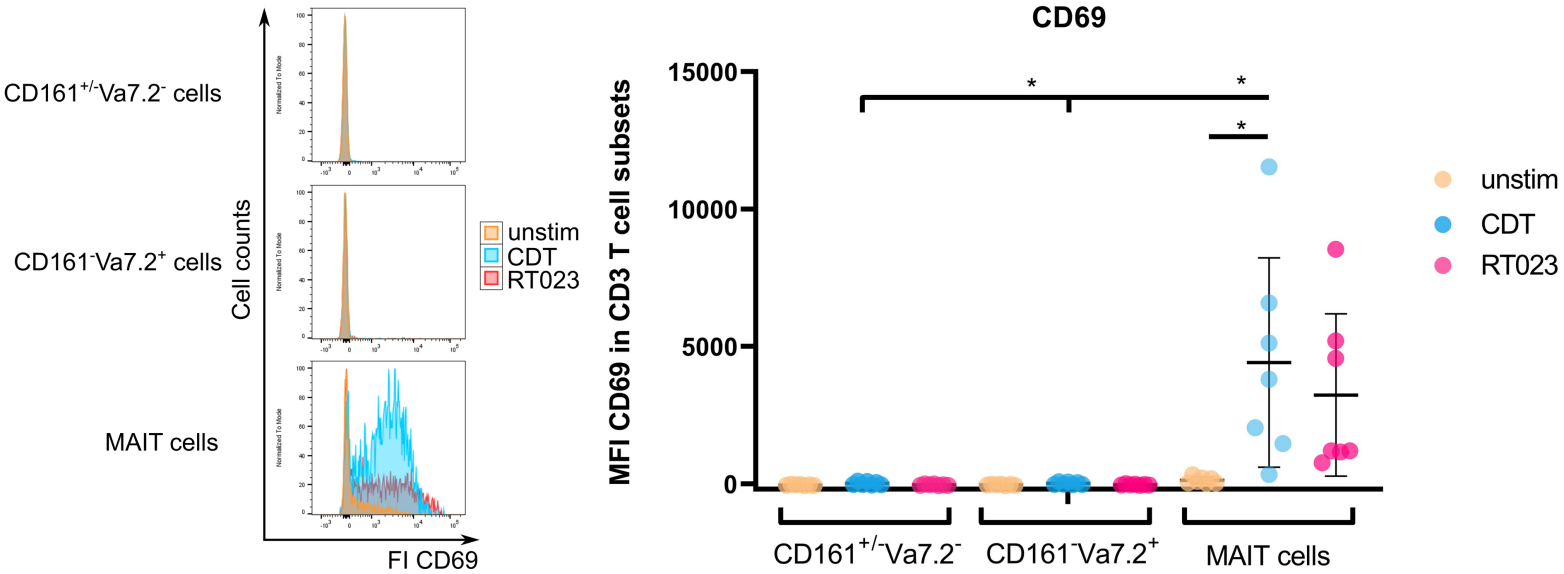
Activation of MAIT cells, CD161^+/−^ Vα7.2^−^, and CD161^−^ Vα7.2^+^ cells following stimulation with *C. difficile* CDT. PBMCs were isolated from healthy donors and stimulated with 100 ng/ml CDT for 20 h followed by flow cytometric analyses of CD69 surface expression. Cells were gated on either CD161^++^ Vα7.2^+^, CD161^+/−^ Vα7.2^−^ or CD161^−^ Vα7.2^+^ CD3^+^ T cells and median fluorescence intensity of CD69 is displayed. Horizontal lines indicate mean ± SD. Asterisks indicate significant differences determined by eight Wilcoxon matched-pairs signed rank tests with Bonferroni Holm correction: *p < 0.05. The following conditions were pairwise compared within all subsets: unstimulated (unstim) vs. CDT or unstim vs. RT023. Additionally, CDT of MAIT cells vs. CDT of CD161^+/−^ Vα7.2^−^ or CD161^−^ Vα7.2^+^ were also compared. Data represent five independent experiments from seven donors.

CDT expression is a hallmark of hypervirulent and most pathogenic *C. difficile* strains that are associated with severe pathogenesis of CDAC. In general, CDT can enhance bacterial adhesion to host cells but its role in T cell-mediated immunity has not been studied so far (Schwan et al., 2009). Since CDT induced a significant MAIT cell activation, we wondered whether this observation is restricted to CD161 and Vα7.2. To evaluate this, PBMCs from healthy individuals were stimulated with CDT as described before, followed by flow cytometric analysis of CD69 on either CD161^++^Vα7.2^+^ (MAIT cells), CD161^−^Vα7.2^+^ or CD161^+/−^ Vα7.2^−^ T cells (Figure 2). In addition, we included stimulation of PBMCs with *C. difficile* RT023 as positive control. High donor variations were observed for stimulation with RT023 and CDT.

Although there was a moderate activation of CD161^−^Vα7.2^+^ and CD161^+/−^ Vα7.2^−^ T cells after stimulation with CDT, MAIT cell activation defined by high expression of CD161 and Vα7.2 was more profound. Moreover, CD161^++^ Vα7.2^+^ MAIT cell activation after CDT stimulation was significantly increased in comparison to CD161^−^Vα7.2^+^ or CD161^+/−^ Vα7.2^−^ T cells. For CD161^−^Vα7.2^+^ T cells, the ΔMFI of CD69 after CDT stimulation was +58.4 and for CD161^+/−^ Vα7.2^−^ T cells +49.9, whereas it was +4286.6 for MAIT cells.

Thus, T cell activation by CDT requires CD161^++^ Vα7.2^+^ suggesting CDT as a potent regulator of MAIT cell-related immune responses.

### CDT-Activated MAIT Cells Exhibit a Cytotoxic Phenotype

**Figure 3:**
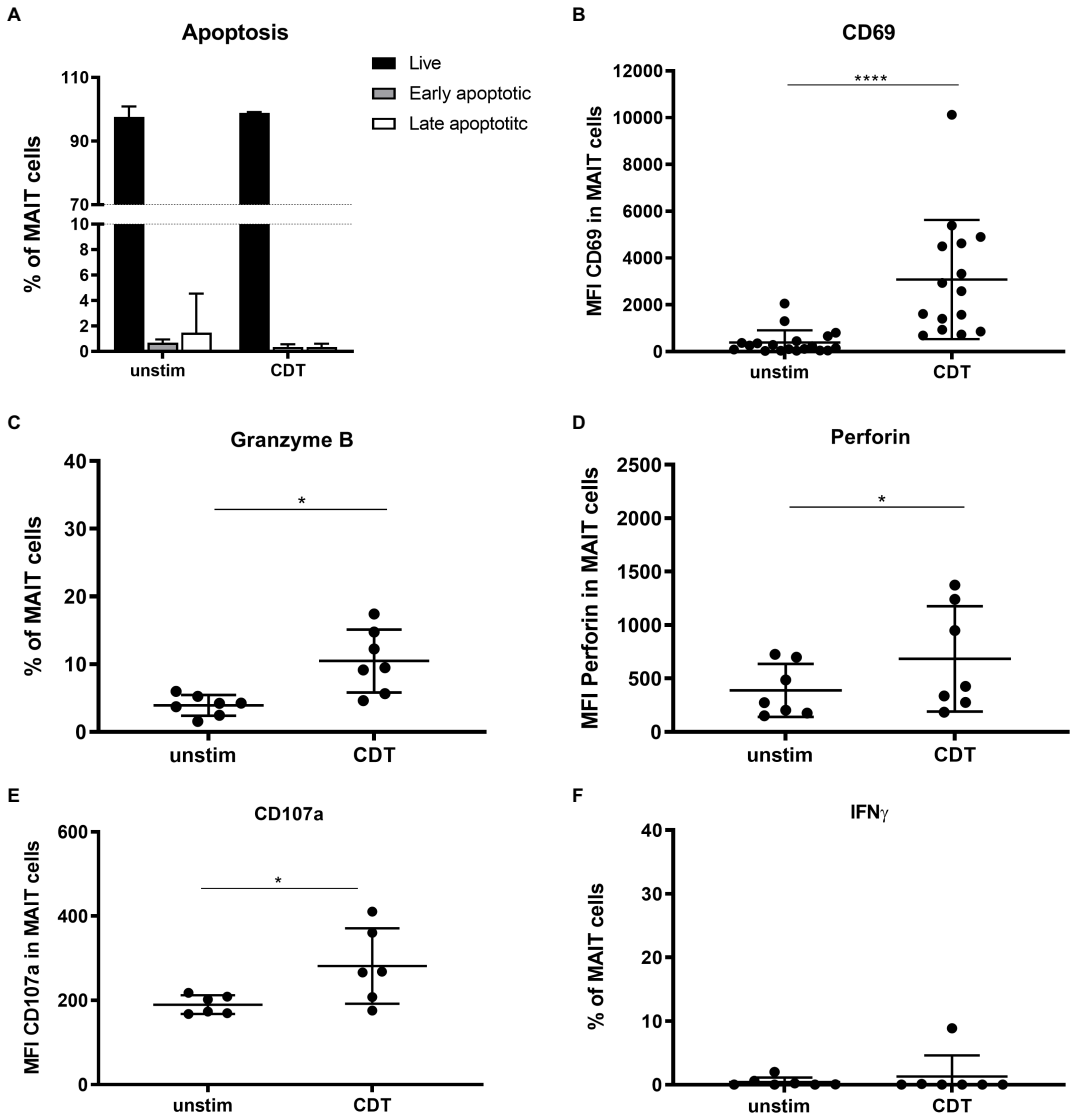
Effector phenotype of primary human MAIT cells following stimulation with *C. difficile* CDT. PBMCs were isolated from healthy donors and stimulated with 100 ng/ml CDT for 20 h followed by flow cytometric analysis of apoptosis **(A)**, granzyme B **(B)**, CD69 **(C)**, perforin **(D)**, CD107a **(E)** and IFNγ expression **(F)**. Cells were gated on CD161^++^ Vα7.2^+^ CD3^+^ T cells (MAIT cells). **(A)** Live: Annexin V^−^ and 7-AAD^−^ (black), early apoptotic Annexin V^+^ and 7-AAD^−^ (dark grey), and late apoptotic Annexin V^+^ and 7-AAD^+^ (white) MAIT cells are depicted. Horizontal lines indicate mean ± SD. Asterisks indicate significant differences determined by Wilcoxon matched-pairs signed rank test: *p < 0.05, ****p < 0.0001. Data represent nine independent experiments from 6 to 15 donors.

Upon activation, MAIT cells mediate effector functions including cytotoxic response and secretion of cytokines. Strong or persistent activation, however, can promote MAIT cell apoptosis as well (Cosgrove et al., 2013; Zhang et al., 2019). Thus, we investigated whether stimulation with *C. difficile* toxin CDT would induce MAIT cell apoptosis, cytotoxicity and/or cytokine expression. As shown before, MAIT cells readily responded to CDT stimulation by significant upregulation of CD69. We then evaluated cell viabilities by assessing live, early and late apoptotic/dead MAIT cells by Annexin-V and 7-AAD staining. We found no evidence that under the chosen experimental conditions CDT induces MAIT cell apoptosis or cell death (Figures 3A,B). However, CDT-activated MAIT cells showed significantly increased expression of lytic granule components granzyme B (GzmB) as well as perforin. Furthermore, CDT-activated MAIT cells were found to upregulate the degranulation marker CD107a at their surface (Figures 3C–E) indicating preceding degranulation and the release of cytotoxic lytic granules. Strikingly, CDT stimulation did not induce IFNγ expression in MAIT cells (Figure 3F). In conclusion, CDT-mediated activation of MAIT cells promotes their cytotoxicity, which is however uncoupled from an inflammatory IFNγ response.

### CDT-Induced MAIT Cell Cytotoxicity Involves MR1 and IL-18

**Figure 4:**
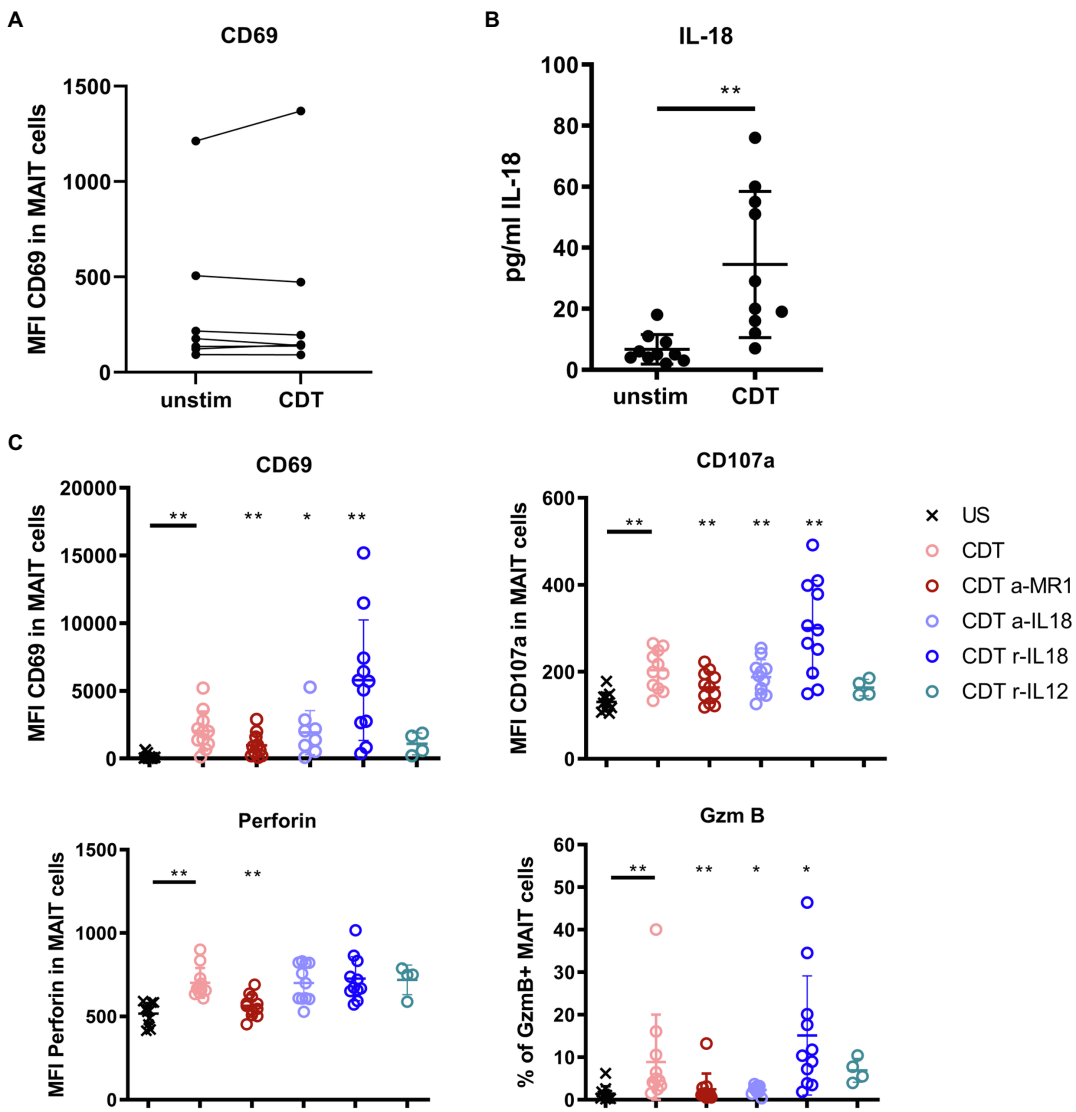
Contribution of IL-18, IL-12, and MR1 to CDT-induced MAIT cell activation and cytotoxicity. *C. difficile* CDT (100 ng/ml) was directly applied to isolated CD161^++^ Vα7.2^+^ CD3^+^ MAIT cells from seven healthy donors for 20 h and MAIT cell activation (CD69) was examined by flow cytometric analyses **(A)**. *C. difficile* CDT (100 ng/ml) was applied to total PBMC fractions from 4 to 11 human donors for 20 h followed by quantification of IL-18 in the PBMC supernatant or flow cytometric analysis of CD69, CD107a, intracellular granzyme B (GzmB), perforin, and IFNγ in MAIT cells **(B,C)**. If indicated, PBMCs were treated with anti-IL-18 (a-IL18), recombinant IL-18 (r-IL18), recombinant IL-12 (r-IL12) or anti-MR1 (a-MR1) prior CDT stimulation. Horizontal lines indicate mean ± SD. Asterisks indicate significant differences determined by four Wilcoxon matched-pairs signed rank tests with Bonferroni Holm correction. Each condition was pairwise compared to CDT alone: *p < 0.05, **p < 0.01. Data represent three independent experiments from 4 to 11 donors. US: unstimulated.

So far, it is unknown whether MAIT cells are direct targets of CDT. To test this, sorted MAIT cells were stimulated with CDT and CD69 surface expression was assessed. The CD69 expression was not increased, i.e., CDT stimulation of purified MAIT cells did not induce their activation, suggesting that previously observed CDT-dependent MAIT cell activation requires accessory cells (Figure 4A). Assuming that CDT affects cells other than MAIT cells in the PBMC fraction, we investigated whether those would release MAIT cell-activating cytokines (IL-12, IL-15, and IL-18) following stimulation with CDT. While IL-12 and IL-15 were not detectable (data not shown), CDT stimulation resulted in increased IL-18 levels in the supernatant of CDT-stimulated PBMCs (Figure 4B). Therefore, we next examined the role of IL-18 for the CDT-dependent MAIT cell activation.

Antibody-mediated blockade of IL-18 responses (a-IL-18) 1 h prior CDT stimulation in PBMC fractions significantly reduced the expression of the cytotoxic granule component GzmB. The expression of CD69 and CD107a was moderately but yet significantly reduced while a-IL-18 treatment did not affect the expression of perforin in MAIT cells (Figure 4C). We furthermore validated the role of IL-18 in the CDT-mediated activation of MAIT cells by the addition of supplementary IL-18 (r-IL18). Interestingly, IL-18 promoted CDT-induced MAIT cell cytotoxicity (significantly increased CD69, CD107a and GzmB expression). In summary, the level of IL-18 was increased in the supernatant of CDT-stimulated PBMCs and this cytokine mostly affected the CDT-induced GzmB responses while its impact on CD69 and CD107a expression was moderate with, so far, non-proven biological relevance.

Next, to activating cytokines, MAIT cell cytotoxicity can be induced by MR1 presentation of antigens to the MAIT cell invariant T cell receptor (TCR). Thus, we examined whether the MR1-TCR axis would as well contribute to the observed CDT-induced MAIT cell activation. We applied an MR1-blocking antibody and the according isotype control prior stimulation with CDT to the PBMCs and assessed MAIT cell activation and effector responses by flow cytometry (Figure 4C and Supplementary Figure 5). Surprisingly, despite the absence of bacterial antigens, antibody-mediated inhibition of MR1 attenuated MAIT cell activation and cytotoxicity (CD69, CD107a, GzmB and perforin expression). In parallel, we observed a minor downregulation of the MAIT TCR Vα7.2 upon CDT-induced activation, which is typically associated with TCR-MHC dependent activation (Supplementary Figure 6). However, the level of downregulation was subtle when compared to stimulation with fixed bacteria such as *E. coli* and validation of TCR-engagement needs further investigation. In contrast, MR1 blockade did not affect activation of CD161^+/−^ Vα7.2^−^ and CD161^−^ Vα7.2^+^ cells (data not shown).

Additionally, we applied the competitive inhibitor Acetyl-6-formylpterin (Ac-6-FP) to PBMCs during CDT stimulation (Supplementary Figure 7). Ac-6-FP is known to be loaded onto MR1 and to effectively reduce MAIT cell activation following treatment with 5-(2-oxopropylideneamino)-6-d-ribitylaminouracil (5-OP-RU; Eckle et al., 2014). Indeed, we observed an inhibitory effect of Ac-6-FP on CDT-mediated MAIT cell activation, which we found moderate though significant for CD69 and GzmB responses, whereas the effect was marginal for perforin and CD107a.

In conclusion, although the biological relevance of IL-18 was not proven in this study, the data indicate that it may promote the release of the granzyme B by MAIT cells upon CDT-stimulation. However, blockade of MR1 significantly inhibited the MAIT cell effector responses, which suggests that CDT-induced cytotoxicity involves the MR1-pathway.

### MAIT Cell Activation by CDT Involves Monocytes

**Figure 5:**
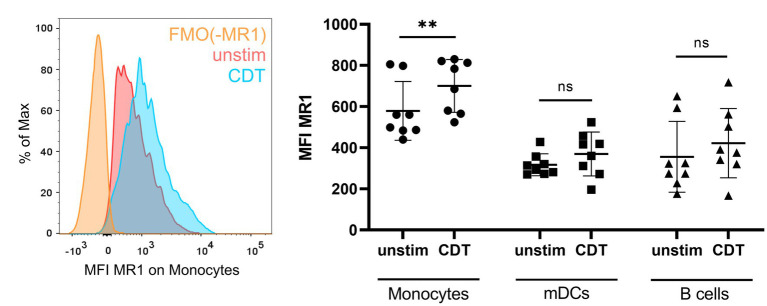
*C. difficile* CDT-induced MR1 surface expression on monocytes. PBMCs were isolated from human donors and either left untreated or stimulated with 100 ng/ml *C. difficile* CDT for 20 h followed by flow cytometric analysis of MR1 expression. Left: Representative histogram of MR1 surface expression (MFI) on unstimulated (red) and CDT-stimulated (blue) CD14^+^ monocytes. Fluorescence minus one (FMO) staining for MR1 is shown (orange). Right: MR1 surface expression of unstimulated or CDT-stimulated CD14^+^ monocytes, myeloid dendritic cells (mDCs), and B cells. Horizontal lines indicate mean ± SD. Asterisks indicate significant differences determined by Wilcoxon matched-pairs signed rank tests: **p < 0.01. Data of two independent experiments from 10 donors are shown.

The fact that CDT-induced MAIT cell responses require MR1 and PBMCs indicates the relevance of antigen-presenting cells that are targeted and modulated by CDT and subsequently activate MAIT cells. To identify those cells, we first monitored MR1 surface expression in PBMCs by flow cytometry using a gating strategy to examine three major MR1-expressing immune cell subsets, i.e., monocytes, myeloid dendritic cells (mDCs) and B cells (Supplementary Figure 8). In the absence of CDT, highest MR1 basal expression was observed on CD14^+^ monocytes (Figure 5, right). While CDT stimulation did not significantly alter MR1 surface expression on mDCs and B cells, a significantly increased MR1 surface expression was found on CD14^+^ monocytes (Figure 5, left and right). These data suggest that CD14^+^ monocytes are targeted by CDT.

**Figure 6:**
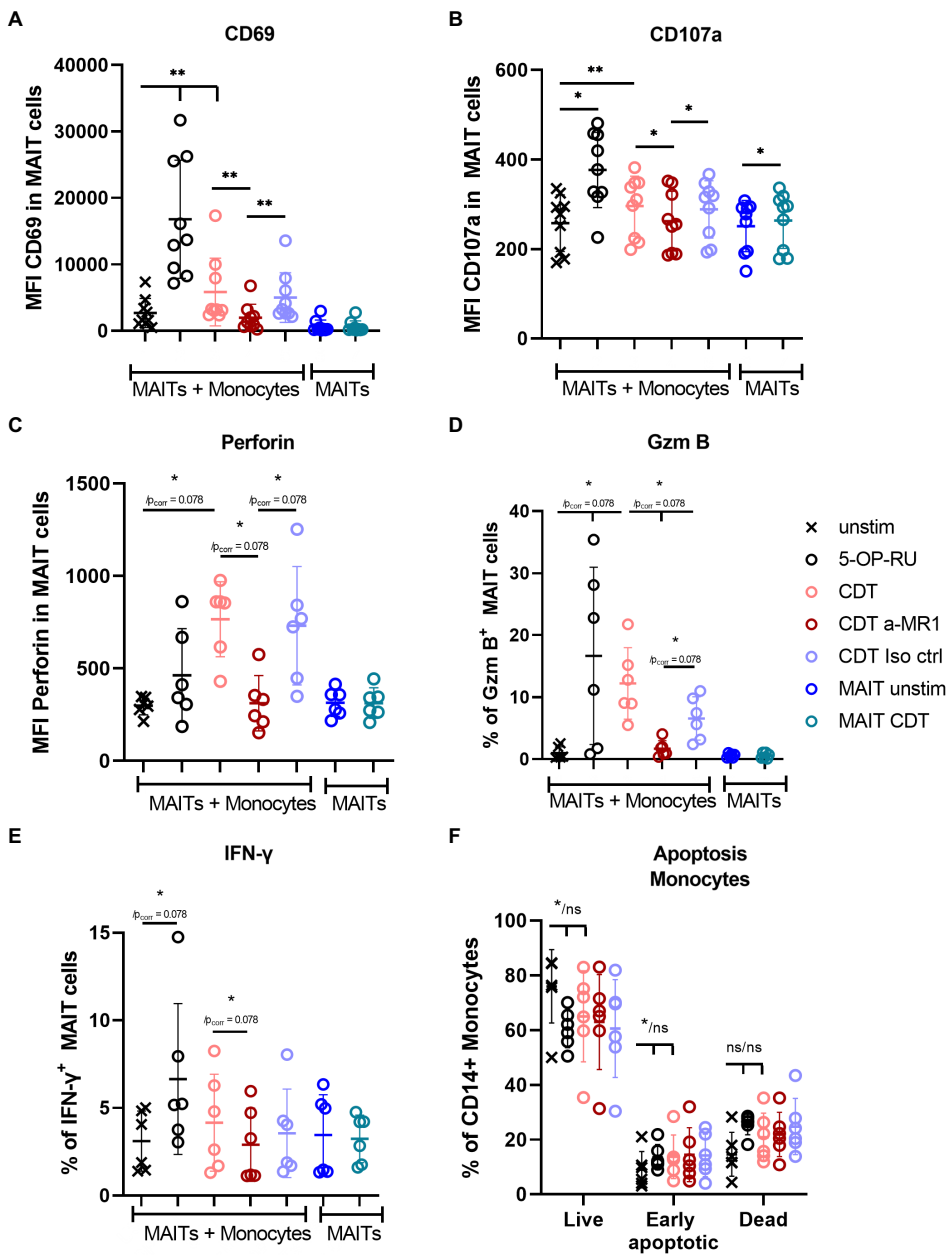
Monocytes MR1-dependently activate MAIT cells in a bi-cellular model following CDT stimulation. *C. difficile* CDT (100 ng/ml) was directly applied to isolated CD161^++^ Vα7.2^+^ CD3^+^ MAIT cells and CD14^+^ monocytes (1:1 ratio) for 20 h. As indicated, in two conditions MAIT cells were stimulated without monocytes. MAIT cell activation was quantified by flow cytometric analysis determining CD69 **(A)**, CD107a **(B)**, intracellular perforin **(C)**, GzmB **(D)** and IFNγ **(E)** expression. Apoptosis of monocytes was determined by Annexin V Apoptosis Detection Kit with 7-AAD **(F)**. If indicated, cells were treated with anti-MR1 (a-MR1) or isotope control IgG1 (Iso ctrl) 1 h prior CDT stimulation. As positive control, cells were stimulated with 50 ng/ml 5-OP-RU for 20 h. Horizontal lines indicate mean ± SD. Asterisks indicate significant differences determined by Wilcoxon matched-pairs signed rank test with *p* values before and after Bonferroni Holm correction (*/p_corr_); *p < 0.05; **p < 0.01. If no corrected p value is given, asterisks indicate significances after the correction. For co-incubated MAIT cells and monocytes (MAITs + Monocytes) five tests were performed **(A–E)**: unstimulated (unstim) vs. (i) 5-OP-RU, (ii) CDT and CDT vs. (iii) a-MR1, (iv) Iso ctrl, and (v) a-MR1 vs. Iso ctrl. MAIT cells alone ± CDT were tested separately. In **(F)** three tests were performed between unstim and indicated conditions. Data of two to three independent experiments from six to nine donors are shown.

Next, we investigated the role of CD14^+^ monocytes during CDT-induced MAIT cell activation by stimulating sorted MAIT cells and sorted monocytes of the same donors in a bi-cellular model with CDT. CDT stimulation of sorted MAIT cells alone had no effect on CD69 surface expression, whereas in the presence of monocytes, CD69 expression was significantly increased (Figure 6A). Furthermore, CDT-activated MAIT cells showed significantly increased expression of CD107a in the bi-cellular model, indicating preceding degranulation. Expression of the lytic granule components GzmB and perforin were also increased after CDT stimulation with monocytes. However, due to relatively low donor numbers and by taking multiple testing into account by the strict Bonferroni Holm method, the *p*-values of increased GzmB and perforin expression were calculated with 0.078. Nevertheless, these data are in accordance with CDT-stimulated PBMCs (Figure 3) suggesting the role of monocytes for CDT-induced MAIT cell activation. We did not observe a significant increase in apoptosis of monocytes after 20 h of CDT stimulation, which largely excludes the possibility that MAIT cell activation and cytokine production may result from CDT-induced necrotic or apoptotic monocytes (Figure 6F). Responsiveness of the donors towards IFNγ response was shown by the positive control 5-OP-RU. IFNγ production was slightly increased in individual donors after CDT stimulation, but we did not observe a significant induction of IFNγ by CDT in the bi-cellular model (Figure 6E). Again, this finding is in agreement with our data from PBMCs (see Figure 3F). Likewise, MR1-dependency was validated in this bi-cellular model, since the application of anti-MR1 antibodies prior to CDT stimulation decreased MAIT cell effector responses (CD69, CD107a, GzmB, perforin).

In summary, CDT can induce MAIT cell activation and cytotoxicity, which was found to depend on MR1. Moreover, MR1-expressing monocytes were sufficient to induce significant MAIT cell activation following CDT stimulation.

### CDT-Expressing Hypervirulent *C. difficile* Enhance MAIT Cell Activation

**Figure 7:**
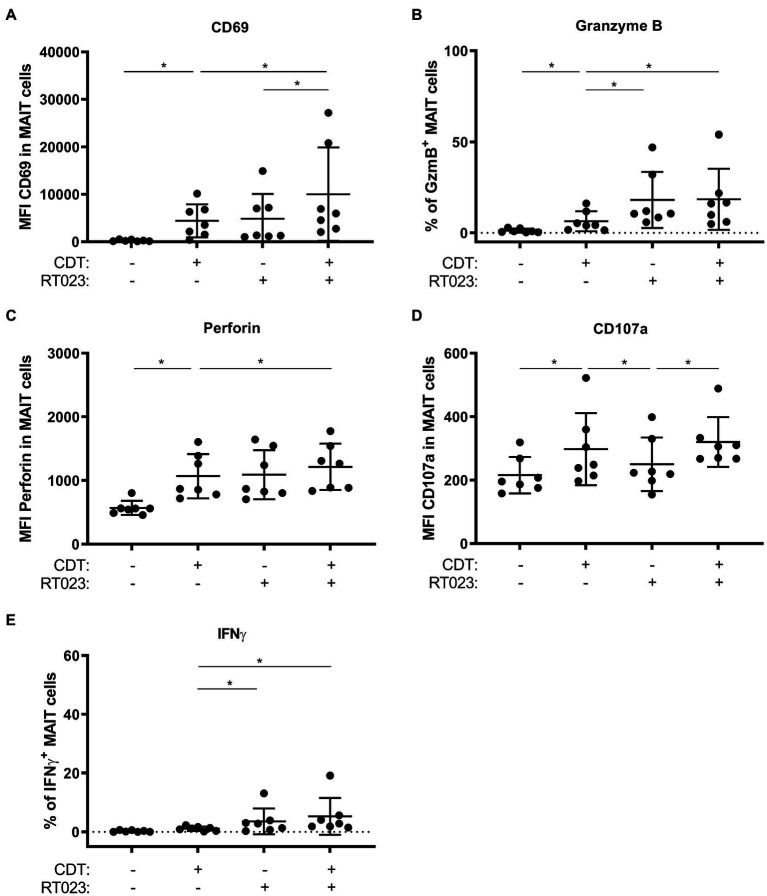
MAIT cell effector phenotype following combined stimulation with hypervirulent *C. difficile* isolate RT023 and/or CDT. PBMCs were isolated from healthy donors and stimulated with *C. difficile* isolate with ribotype RT023 at MOI 1 and/or 100 ng/ml CDT for 20 h followed by flow cytometric analyses of CD69 **(A)** and CD107a **(D)** surface expression, internal granzyme B **(B)**, perforin **(C)**, and IFNγ **(E)** expression. Cells were gated on CD161^++^ Vα7.2^+^ CD3^+^ T cells (MAIT cells). Horizontal lines indicate mean fluorescence intensity/mean percentages ± SD. Asterisks indicate significant differences determined by six Wilcoxon matched-pairs signed rank tests with Bonferroni Holm correction: *p < 0.05. All conditions were pairwise tested against each other. Data of three independent experiments from seven donors are shown.

We have previously shown that hypervirulent *C. difficile* ribotypes, which under the chosen cultivation conditions did not express toxins, induce MAIT cell responses MR1-dependently (Bernal et al., 2018). Here, we demonstrate that *C. difficile* toxin CDT induces cytotoxic effector functions as well in an MR1-dependent manner. Thus, we next analyzed whether simultaneous stimulation of MAIT cells with (i) the hypervirulent *C. difficile* ribotype RT023 and (ii) CDT would have a synergistic effect in terms of MAIT cell activation and their acquisition of effector functions. To this end, PBMCs from healthy individuals were stimulated according to the previously established conditions using PFA-fixed *C. difficile* RT023 at an MOI 1 (Bernal et al., 2018), with 100 ng/ml CDT, or a combination of both. As expected, both conditions alone resulted in the activation of MAIT cells. Interestingly, co-stimulation with RT023 and CDT further increased the MAIT cell activation in individual donors as indicated by enhanced CD69 surface expression compared to stimulation with RT023 or CDT only (Figure 7A). However, co-stimulation with CDT did not induce further upregulation of the *C. difficile* RT023-induced expression of cytotoxic GzmB and perforin (Figures 7B,C). CDT-induced CD107a expression was not further increased by co-stimulation with *C. difficile* RT023 (Figure 7D). Likewise and in accordance with the previously described data, CDT did not further increase the *C. difficile* RT023-induced IFNγ response (Figure 7E). In conclusion, co-stimulation with CDT synergizes with *C. difficile*-induced MAIT activation (CD69), while it does not further boost *C. difficile*-induced upregulation of cytotoxic perforin and GzmB as well as inflammatory IFNγ responses.

## Discussion

Virulent *C. difficile* is capable to produce three different exotoxins which, following receptor binding and uptake, manipulate host cell processes: TcdA, TcdB and *C. difficile* transferase CDT. The exotoxins TcdA and TcdB that contain glycosylation and ribosylation enzymatic domains inactivate Rho GTPases thereby causing *C. difficile*-associated colitis (CDAC). Hypervirulent strains additionally can produce the binary, actin ADP-ribosylating toxin CDT and these strains are associated with severe CDAC progression including pseudomembranous colitis, loss of intestinal barrier functions and life-threatening sepsis.

We show here that toxins of hypervirulent *C. difficile* are capable to activate MAIT cells in PBMC fractions of healthy individuals. In case of TcdA and TcdB, MAIT cell activation following stimulation with glycosyltransferase-deficient mutants was reduced in comparison to enzymatically active TcdA and TcdB, indicating a role of Rho GTPase signaling in this process. However, CD69 expression was analyzed from two different groups of donors in Supplementary Figure 4, limiting the possibility to make a final conclusion about the enzymatic dependency.

In contrast, CDT, which is only expressed by hypervirulent *C. difficile* strains, seems to activate MAIT cells in a different manner. Binary CDT consists of a substrate binding and pore-forming CDTb and an ADP-ribosyl-transferase containing CDTa domain. ADP-ribosylation of actin by CDT causes depolymerization of the host cytoskeleton and the formation of microtubule-rich protrusions, which support bacterial adhesion (Schwan et al., 2009). Interestingly, activation of MAIT cells was found to be mostly dependent on CDTb, while CDTa and its ADP-ribosyltransferase seemed to be dispensable for MAIT cell activation. Thus, CDT apparently utilizes a mechanism distinct from TcdA and TcdB to activate MAIT cell immunity. Indeed, it was previously reported that *C. difficile* CDTb is sufficient to induce necrosis in epithelial cells indicating signaling competence of the binding and pore-forming part of CDT (Landenberger et al., 2021).

In general, it was surprising that the mode-of-action of CDT in PBMCs required MR1. The prototypic MR1 antibody which is routinely used to characterize MR1-dependency almost completely blocked CDT-mediated MAIT cell responses, suggesting a CDT/MR1 non-canonical pathway being involved in the activation of MAIT cells (Huang et al., 2009). This pathway likely requires MR1 expressing non-MAIT cells since (i) MAIT cells alone turned out to be resistant against *C. difficile* toxin-induced apoptosis (Figure 3A) and (ii) MAIT cells were only activated CDT-dependent as part of PBMC fractions, whereas purified MAIT cells did not up-regulate CD69 following CDT treatments (Figures 4A, 6A). Characterizing MR1-surface expression on PBMCs indeed revealed that CDT triggers the upregulation of MR1 on monocytes and bi-cellular stimulation assays with primary MAIT cells and monocytes verified that monocytes alone are sufficient to mediate CDT-dependent MAIT cell activation. This of course did not rule out the possibility that other cell types contribute to CDT-mediated MAIT cell responses *in vivo*.

The key question by which mechanism CDT provokes MR1-dependent MAIT cell responses was not addressed and requires further studies. Lipolysis-stimulated lipoprotein receptor (LSR) is the main receptor of CDT, which is highly expressed on colonic epithelial cells and is important for the integrity of epithelial barriers (Masuda et al., 2011). In general, immune cells have a relatively low expression of LSR compared to epithelial cells^1^ with decreasing amounts from T cell subsets to antigen-presenting cells. In the same line, one could also speculate about a potential role of toll-like receptor signaling since CDT is recognized by the TLR2/6 heterodimer to induce an NF-κB response and, TLR signaling in human antigen-presenting cells regulates MR1-dependent activation of MAIT cells (Ussher et al., 2016; Simpson et al., 2020). In conclusion, a mode-of-action of CDT *via* LSR is mechanistically counterintuitive due to its low surface expression on immune cells, whereas TLR signaling may co-regulate MR1 on monocytes.

Because bacterial metabolites were absent during *in vitro* CDT stimulation, we may speculate that MR1 might present endogenous metabolites (self-antigens) that are released during CDT-induced host cell modifications or necrosis. It has been described that in addition to bacterial metabolites MR1 can present non-bacterial antigens and self-antigens (Keller et al., 2017; Lepore et al., 2017). However, this has not yet been investigated in the context of *C. difficile* toxin CDT. Such a mechanism would represent a so far undescribed non-canonical pathway enabling toxin-stimulated cells to present self-antigens MR1-dependently, thereby acting as kind of danger signals in order to induce MAIT cell cytotoxicity. Interestingly, in our experimental setting, cytotoxicity was found to depend on IL-18 (except for perforin). In direct contrast, in the canonical pathway bacteria-stimulated MAIT cells express CD107a, perforin, and GzmB independently of IL-18 (Kurioka et al., 2015). Moreover, CDT did not induce IFNγ expression in MAIT cells. Regarding IFNγ induction, one has to consider the TCR- and the cytokine-dependency of MAIT cell activation. CDT induced only IL-18, which alone is not sufficient to induce IFNγ response in MAIT cells (Ussher et al., 2014). However, in case of canonical activation pathway of bacterial antigens, TCR-dependent MAIT cell activation results in IFNγ expression, which was not observed in this study. Therefore, the absence of IFNγ production indicates a TCR-independent mechanism underlying the CDT-induced MAIT cell activation. Besides, the absence of an IFNγ response largely excludes the possibility that MAIT cells might be activated by contaminants like LPS in the recombinant produced CDT fractions and underlines that CDT specifically activates MAIT cells. Furthermore, the observed minor downregulation of the TCR on MAIT cells, which is notably low as compared to *E. coli* stimulated MAIT cells, did not indicate a significant role of the TCR in the process of CDT-induced responses (Supplementary Figure 6). Thus, we rather suggest a CDT mechanism widely independent from the engagement of the TCR by MR1.

Data of this study now suggest that CDT triggers a non-canonical host cell immune response that ultimately results in the degranulation of cytotoxic granules by MAIT cells. Consequently, MAIT cells might be primed to elicit targeted killing of CDT-intoxicated cells in the blood circulation, thereby contributing to clearance of toxemia. Assuming that MAIT cells exhibit cytotoxicity against CDT intoxicated epithelial cells in CDI, they might as well enhance the CDT-induced disruption of the intestinal barrier and thereby contribute to pronounced immunopathology caused by hypervirulent *C. difficile*. Other studies that investigated MAIT cell activation by Superantigens (SAgs) produced by *Staphylococcus aureus* and *Streptococcus pyogenes,* described an MR1-independent mechanism (Shaler et al., 2017; Emgård et al., 2019). However, they identified MAIT cells as the major responders of SAgs by releasing pro-inflammatory cytokines (Shaler et al., 2017). Furthermore, Emgård et al. suggest that MAIT cells may contribute to the pathological cytokine storm underlying the Streptococcal toxic shock syndrome (STSS) which is similar to our suggestion considering the pronounced immunopathology caused by hypervirulent *C. difficile.*

In general, CDT might be a promising target to manipulate the host cell immune response in CDI caused by hypervirulent *C. difficile*. Our findings suggest that CDTb is the key component of the binary CDT that induces host cell immune responses and in turn provokes MAIT cell cytotoxicity. In this context, neutralizing antibodies against CDTb have been demonstrated to reduce the adherence of *C. difficile* to human colon carcinoma cells (Schwan et al., 2009). Nevertheless, results of our study do not rule out the possibility that CDT affects also other T cell subsets, which might also contribute to pathogenicity during CDI. Thus, inhibiting MAIT cell activation by CDTb neutralizing antibodies might only partially enable to manipulate host cell immune responses during CDI. Of note, CDT-induced MAIT cell activation uncovered a remarkable donor variation concerning the individual strength of effector molecule expression. In particular, it would be important to validate these effector responses at sides of infection to clarify whether anti-CDT strategies support a required MAIT cell immunity.

In conclusion, we discovered a CDT/MR1-axis constituting a non-canonical pathway that selectively induces MAIT cell cytotoxicity. Based on our data, it is tempting to speculate that CDT expression and related cytotoxicity is instrumental for hypervirulent *C. difficile* to overcome epithelial barriers at the intestinal mucosa.

## Data Availability Statement

The raw data supporting the conclusions of this article will be made available by the authors, without undue reservation.

## Ethics Statement

The studies involving human participants were reviewed and approved by Institutional Review Board of the Hanover Medical School. The patients/participants provided their written informed consent to participate in this study.

## Author Contributions

DB and LJ conceived and designed the research. IM, JJ, and JS designed and performed the experiments and analyzed the data. JH and MN-S provided the bacteria. RG provided the toxins. FK designed and performed the statistical analyses. IM, JJ, DB, and LJ wrote the manuscript. All authors contributed to the article and approved the submitted version.

## Funding

This work was conducted within the context of the International Graduate School ABINEP (Analysis, Imaging, and Modelling of Neuronal and Inflammatory Processes) at Otto von Guericke University (OVGU) Magdeburg, Germany. The project was funded by the federal state Saxony-Anhalt and the European Structural and Investment Funds (ESF, 2014-2020), project number ZS/2016/08/80645. to IM and JJ, and by the Federal State of Lower Saxony, Niedersächsisches Vorab (VWZN2889/3215/3266).

## Conflict of Interest

The authors declare that the research was conducted in the absence of any commercial or financial relationships that could be construed as a potential conflict of interest.

## Publisher’s Note

All claims expressed in this article are solely those of the authors and do not necessarily represent those of their affiliated organizations, or those of the publisher, the editors and the reviewers. Any product that may be evaluated in this article, or claim that may be made by its manufacturer, is not guaranteed or endorsed by the publisher.

## Footnotes

^1^ https://www.proteinatlas.org/
