# Supplementary material for: Clostridioides difficile Toxin CDT Induces Cytotoxic Responses in Human Mucosal-Associated Invariant T (MAIT) Cells
Source: Front Microbiol. 2021 Dec 21;12:752549. doi: 10.3389/fmicb.2021.752549 (PMC8727052; doi:10.3389/fmicb.2021.752549)
Supplement: Supplementary file 1 [file Data_Sheet_1.pdf]

Supplementary material

Clostridioides difficile toxin CDT reveals non-canonical MR1-pathway that induces cytotoxic responses in human mucosal-associated invariant T (MAIT) cells

Isabel Marquardt[†,1,2,3], Josefine Jakob[†,1,2,3], Jessica Scheibel[1], Julia Danielle Hofmann[4], Frank Klawonn[1], Meina Neumann-Schaal[4,5], Ralf Gerhard[6], Dunja Bruder[2,3,*†], Lothar Jänsch[1,*†]

[*†]Co-corresponding authors:
Dunja Bruder: dunja.bruder@med.ovgu.de
Lothar Jänsch: lothar.jaensch@helmholtz-hzi.de

Supplementary figures

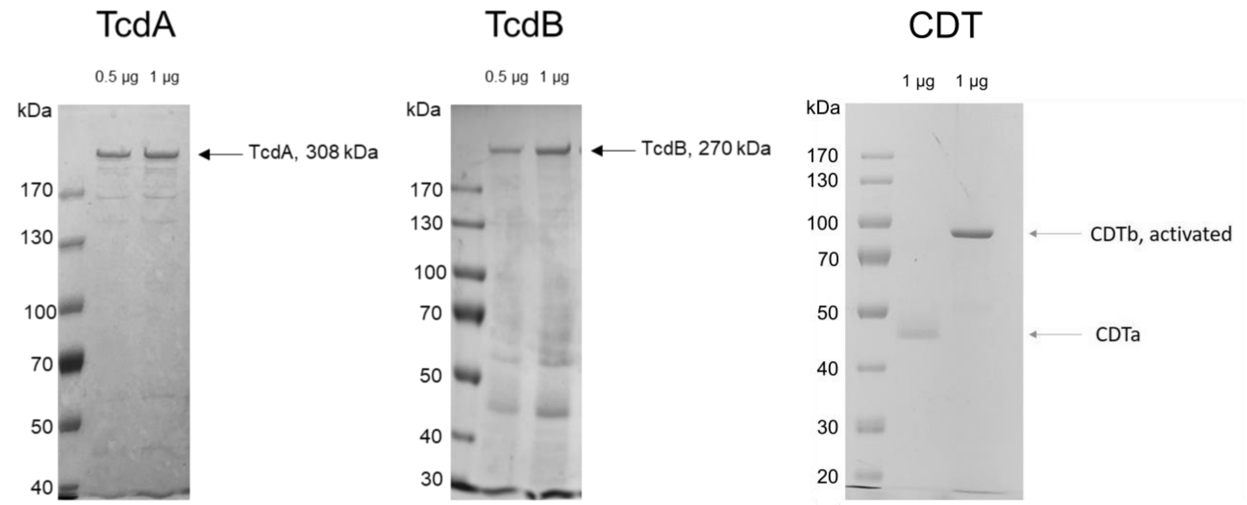

Supplementary figure 1: Coomassie-stained SDS gels of purified *C. difficile* toxins. *C. difficile* toxin TcdA and TcdB were produced in Gram positive and LPS-free *Bacillus megaterium*. *C. difficile* toxin CDT components CDTa and CDTb were produced in *E. coli*. Purity of TcdA, TcdB, CDTa, and CDTb was controlled by SDS-Page following Coomassie staining. 7.5 % acrylamide gel was used for TcdA and TcdB and a 10 % acrylamide gel for CDTa and CDTb. PageRuler™ prestained protein ladder was used as standard molecular weight marker. Expected bands for respective *C. difficile* toxin are indicated. Representative gels are shown for one production batch of TcdA and TcdB, and CDTa and CDTb.

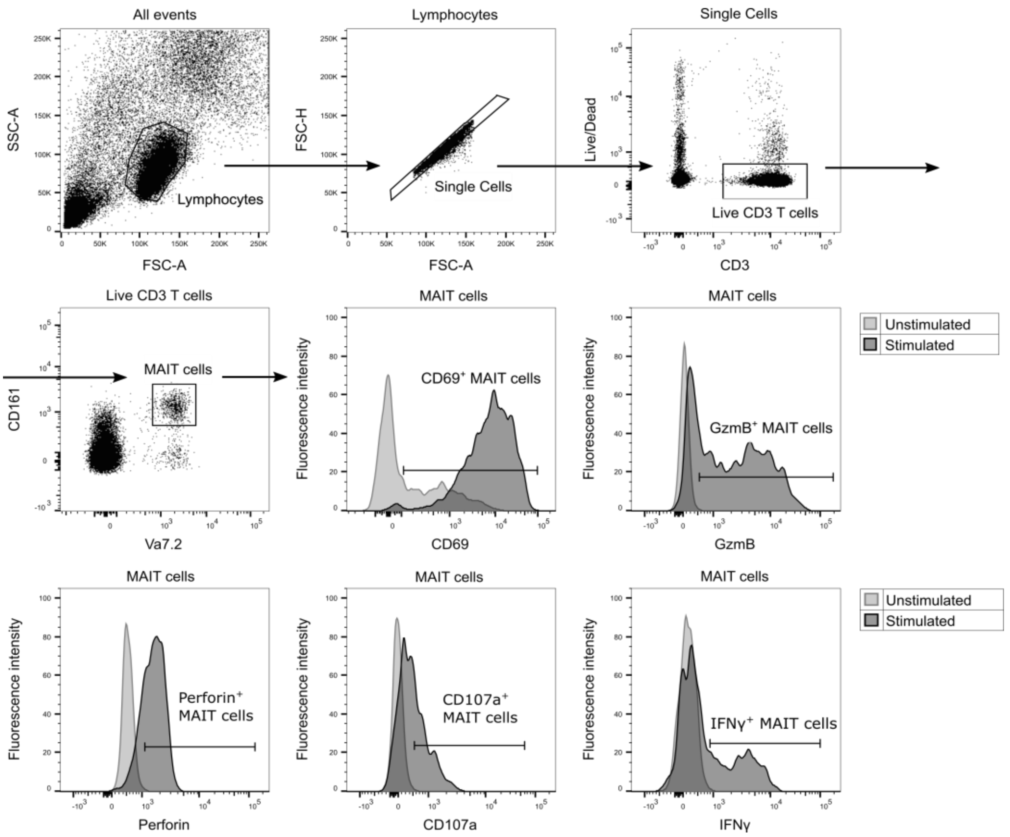

Supplementary figure 2: Gating strategy for human mucosal-associated invariant T (MAIT) cells. PBMCs were isolated from human blood; live cells were discriminated with Live/Dead dye. Cells were also stained with antibodies specific for CD3, Vα7.2, CD161, CD69, Interferon γ, perforin, granzyme B (GzmB), and CD107a followed by flow cytometric analysis.

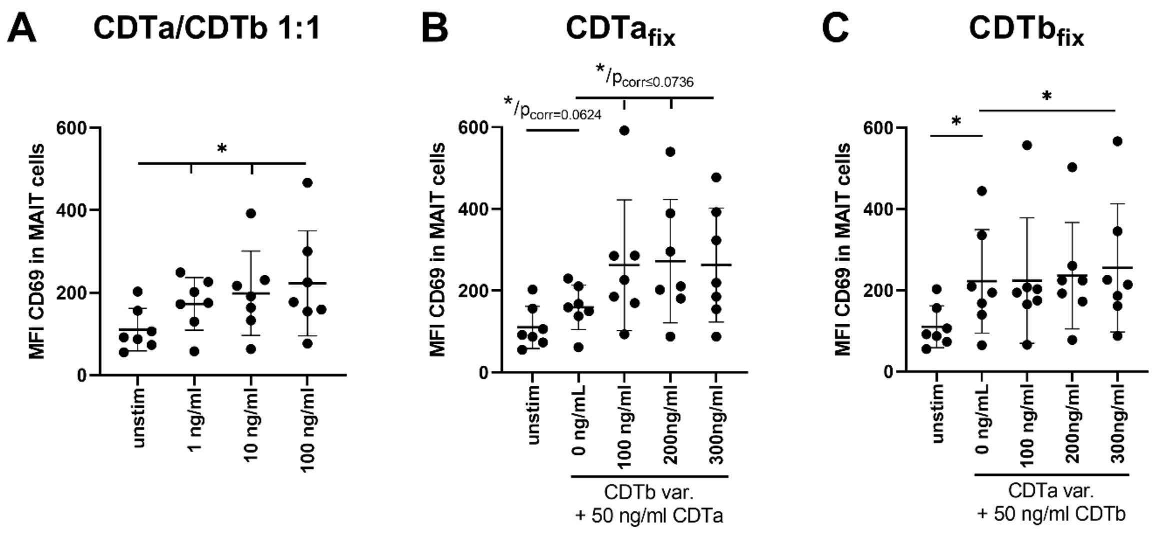

Supplementary figure 3: CDT-induced MAIT cell activation. PBMCs were isolated from healthy donors and stimulated with increasing concentrations *C. difficile* CDT (with CDTa and CDTb 1:1) for 20 h followed by flow cytometric analysis of CD69 expression (A). PBMCs were treated with 50 ng/ml CDTa and increasing concentrations of CDTb as indicated (B) or vice versa (C). MAIT cells were gated as $CD161^{++}$ $V\alpha7.2^{+}$ $CD3^{+}$ T cells. Data represent three independent experiments from eight donors. Horizontal lines indicate mean ± SD. Asterisks indicate significant differences (*$p < 0.05$) as determined by Wilcoxon matched-pairs signed rank test with Bonferroni Holm correction. The following statistical comparisons have been performed: (A) three statistical tests between unstimulated (unstim) and the three different concentrations; (B+C) four tests between 0 ng/ml and all other conditions. Bonferroni Holm corrected p value is shown except for (B) were p values before and after correction are given.

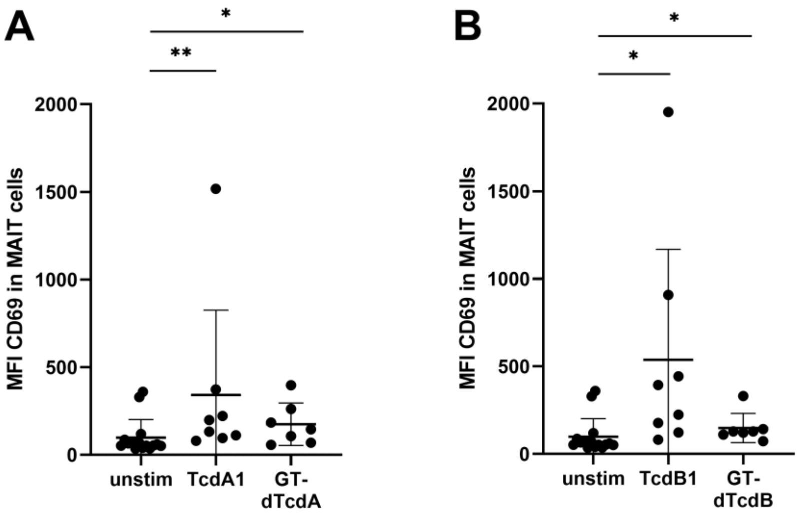

Supplementary figure 4: TcdA- and TcdB-induced MAIT cell activation. PBMCs were isolated from healthy donors and stimulated with 100 ng/ml of two batches *C. difficile* toxins: (A) TcdA1, TcdA2, and glycosyltransferase-deficient TcdA (GT-dTcdA) and (B) TcdB1, TcdB2, and glycosyltransferase-deficient TcdB (GT-dTcdB) for 20 h followed by flow cytometric analysis of CD69 expression. MAIT cells were gated on $CD161^{++}$ $V\alpha7.2^{+}$ $CD3^{+}$ T cells. Data represent two independent experiments from seven to eight donors. Horizontal lines indicate mean ± SD. Asterisks indicate significant differences (*$p < 0.05$, **$p < 0.01$) as determined by two Wilcoxon matched-pairs signed rank tests between unstimulated (unstim) and stimulated conditions with Bonferroni Holm correction. TcdA1 and GT-dTcdA were analyzed from different donors and compared by Mann-Whitney test ($p = 0.3063$) as well as TcdB1 and GT-dTcdB ($p = 0.0334$).

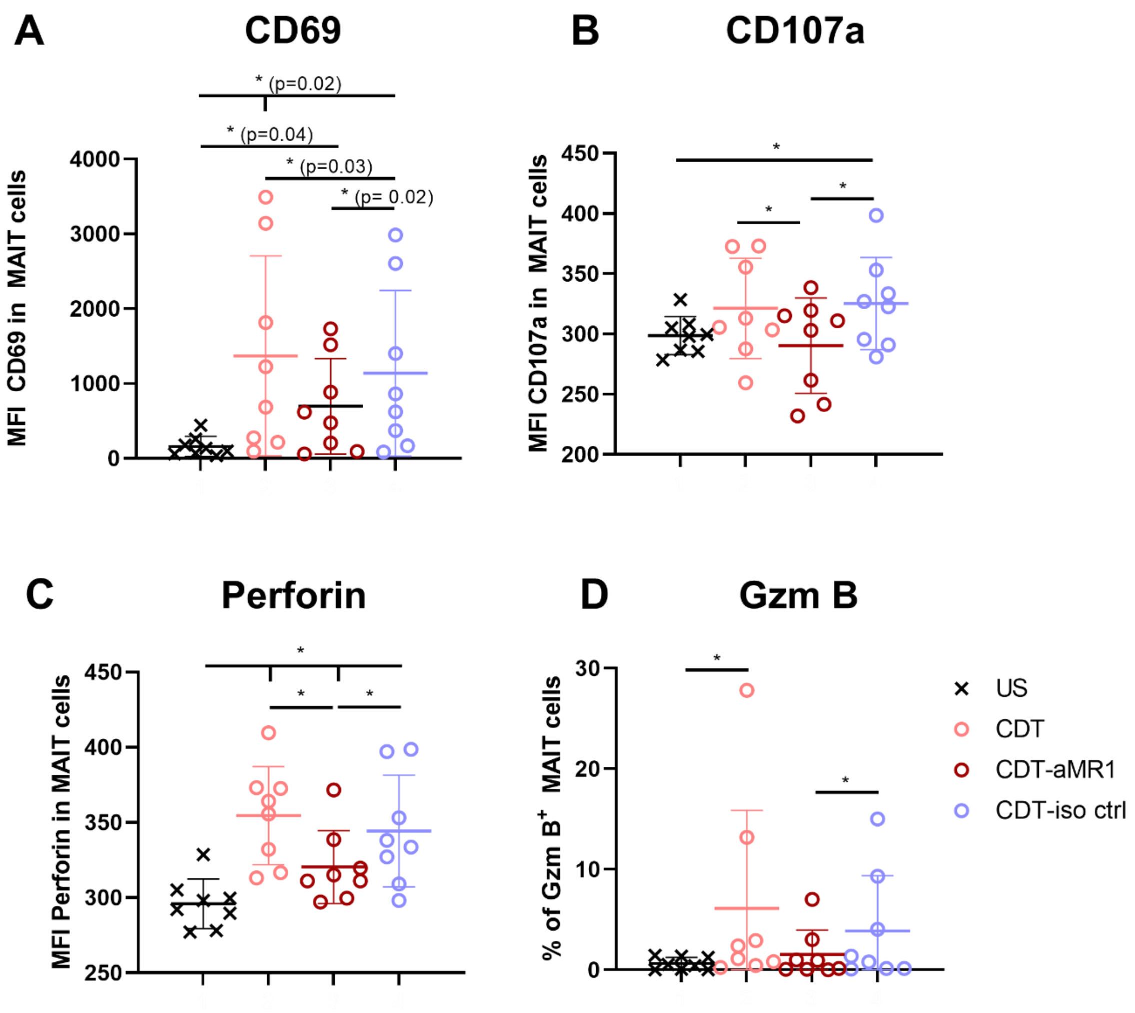

Supplementary figure 5: Contribution of MR1 to CDT-induced MAIT cell activation and cytotoxicity. *C. difficile* CDT (100 ng/ml) was applied to total PBMC fractions from human donors for 20 h followed by flow cytometric analysis of CD69, CD107a, intracellular granzyme B and perforin in MAIT cells (A-D). If indicated, PBMCs were treated with anti-MR1 (a-MR1) or IgG isotype control prior CDT stimulation. Horizontal lines indicate mean ± SD. Asterisks indicate significant differences determined by six Wilcoxon matched-pairs signed rank tests with Bonferroni Holm correction. All conditions were pairwise compared to each other: *p < 0.05. Data represent two independent experiments from eight donors which are different from donors used in Figure 4. US: unstimulated.

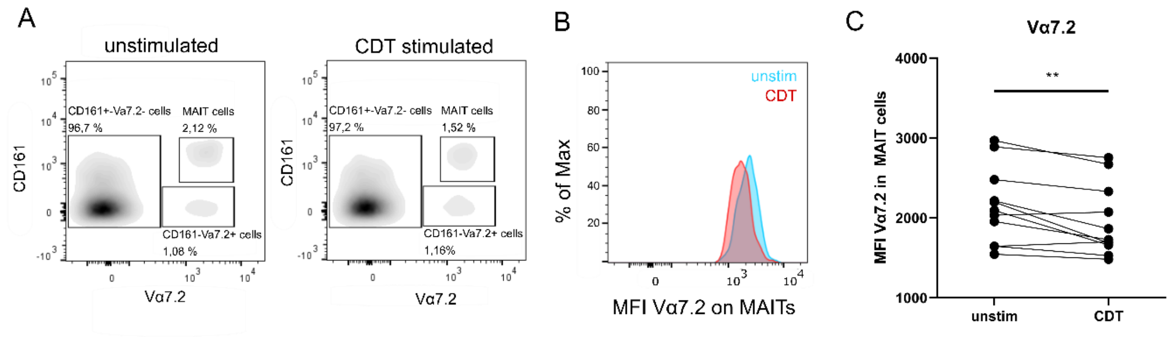

Supplementary figure 6: Surface expression of MAIT TCR Vα7.2 during CDT stimulation. PBMCs were isolated from healthy donors and stimulated with 100 ng/ml CDT for 20 h followed by flow cytometric analysis. Cells were gated on $CD161^{++}$ $V\alpha7.2^{+}$ $CD3^{+}$ T cells (MAIT cells). A) The gating strategy is shown for MAIT cells, $CD161^{+-}V\alpha7.2^{-}$ and $CD161^{-}V\alpha7.2^{+}$ cells ± stimulation with CDT. B) Representative histogram of Vα7.2 surface expression on unstimulated (blue) and CDT-stimulated (red) MAIT cells is shown. C) Median fluorescence intensity (MFI) is shown for Vα7.2 on MAIT cells. Asterisks indicate significant difference determined by Wilcoxon matched-pairs signed rank test: **$p < 0.01$. Data represent three independent experiments from 11 donors. Same donor samples as shown in Figure 4 were analyzed.

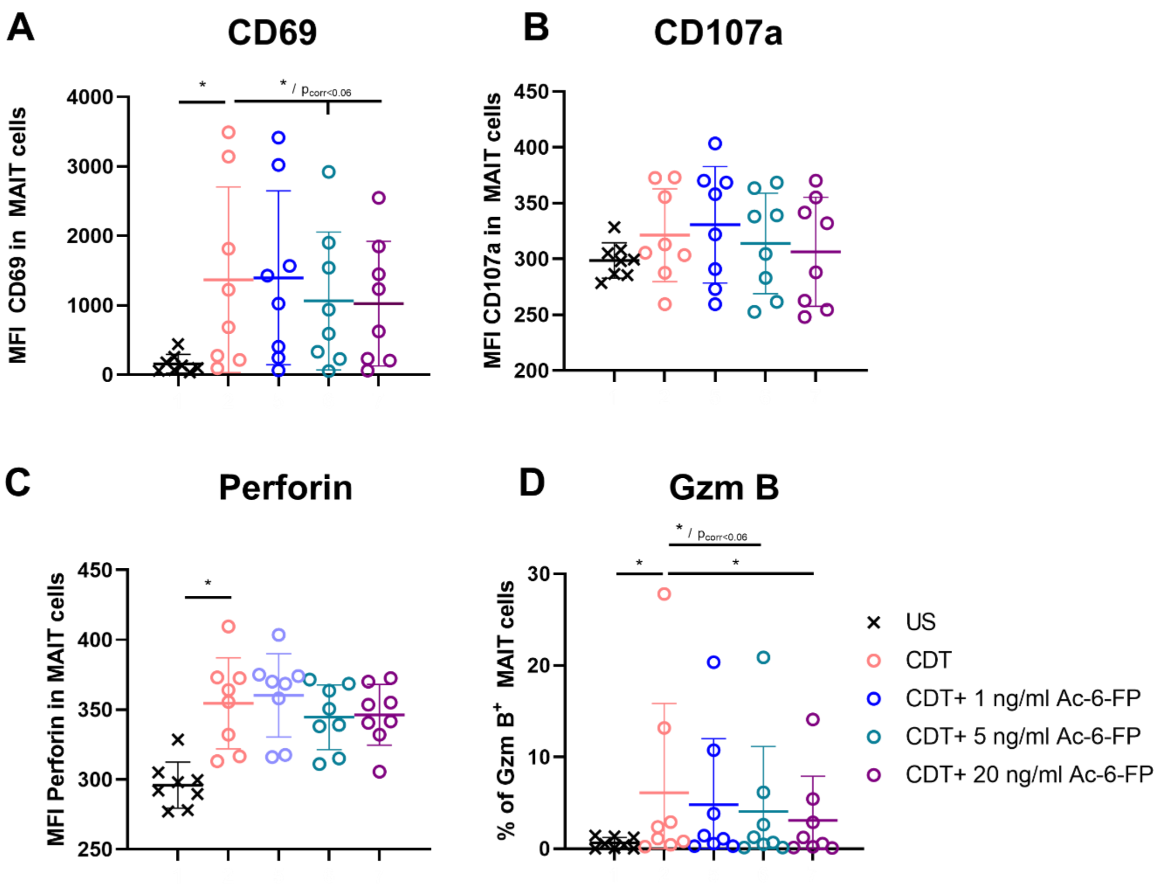

Supplementary figure 7: Inhibition of CDT-induced MAIT cell activation by Ac-6-FP. *C. difficile* CDT (100 ng/ml) was applied to total PBMC fractions from human donors for 20 h followed by flow cytometric analysis of CD69, CD107a, intracellular granzyme B (Gzm B) and perforin in MAIT cells (A-D). If indicated, PBMCs were treated with Ac-6-FP 1 h prior CDT stimulation. Horizontal lines indicate mean ± SD. Asterisks indicate significant differences determined by four Wilcoxon matched-pairs signed rank tests before and after Bonferroni Holm correction (*/p_{corr}); p* < 0.05. If no corrected p value is given, asterisks indicate significances after the correction. Each condition was pairwise compared to CDT alone. Data represent two independent experiments from eight donors. US: unstimulated.

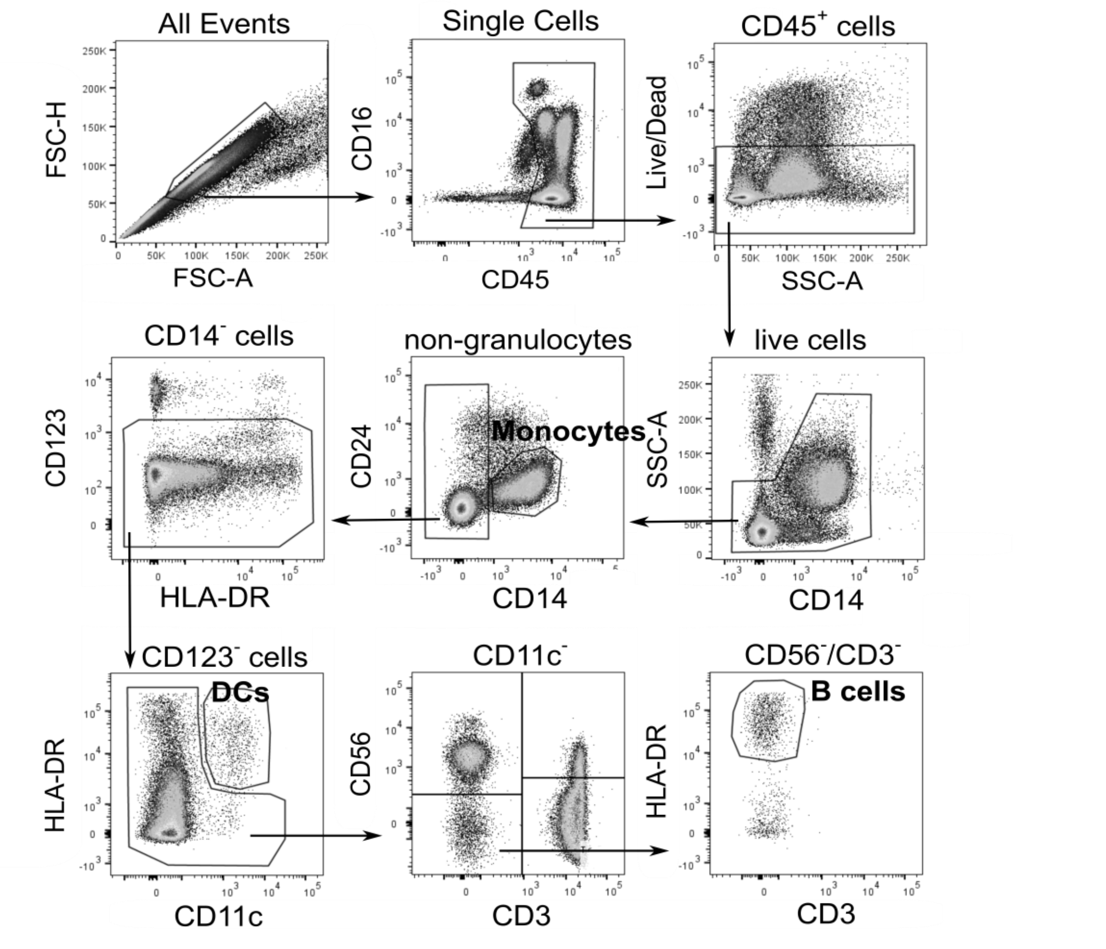

Supplementary figure 8: Gating strategy for monocytes, dendritic cells (DCs), and B cells. PBMCs were isolated from human blood. Single cells were discriminated by forward scatter-area (FSC-A) and forward scatter-height (FSC-H). Live leukocytes ($CD45^+$) cells were discriminated with Live/Dead dye and also stained with antibodies against CD16, CD14, CD24, HLA-DR, CD123, CD11c, CD56, and CD3 followed by flow cytometric analysis.
